# Selection and validation of reference genes by RT-qPCR under photoperiodic induction of flowering in sugarcane (*Saccharum* spp.)

**DOI:** 10.1038/s41598-021-83918-2

**Published:** 2021-02-25

**Authors:** Paulo H. da Silva Santos, João R. Vieira Manechini, Michael S. Brito, Elisson Romanel, Renato Vicentini, Maximiliano Scarpari, Stephen Jackson, Luciana R. Pinto

**Affiliations:** 1 Faculdade de Ciências Agrárias e Veterinárias, UNESP, Campus Jaboticabal, Jaboticabal, CEP14884-900 Brazil; 2 Universidade Estadual de Campinas, UNICAMP, Campinas, CEP13083-970 Brazil; 3 Instituto de Ciência e Tecnologia da Universidade Federal de São Paulo, São José dos Campos, CEP12231-280 Brazil; 4 Departamento de Biotecnologia, Escola de Engenharia de Lorena/Universidade de São Paulo, EEL/USP, Lorena, SP CEP12602-810 Brazil; 5 Instituto Agronômico de Campinas, Centro de Cana, Ribeirão Preto, CEP14032-800 Brazil; 6 School of Life Sciences, University of Warwick, Gibbet Hill, Coventry, CV4 7AL UK

**Keywords:** Gene expression analysis, Biological techniques, Molecular biology

## Abstract

Although reference genes have previously been used in the expression analysis of genes involved in sugarcane flowering they had not been experimentally validated for stability and consistency of expression between different samples over a wide range of experimental conditions. Here we report the analysis of candidate reference genes in different tissue types, at different temporal time-points, in both short and long day photoperiodic treatments. The stability of the candidate reference genes in all conditions was evaluated with NormFinder, BestKeeper, and RefFinder algorithms that complement each other for a more robust analysis. As the Normfinder algorithm was more appropriate for our experimental conditions, greater emphasis was placed on Normfinder when choosing the most stable genes. *UBQ1* and *TUB* were shown to be the most stable reference genes to use for normalizing RT-qPCR gene expression data during floral induction, whilst *25SrRNA1* and *GAPDH* were the least stable. Their use as a reference gene pair was validated by analyzing the expression of two differentially expressed target genes (*PIL5* and *LHP1*). The *UBQ1/TUB* reference genes combination was able to reveal small significant differences in gene expression of the two target genes that were not detectable when using the least stable reference gene combination. These results can be used to inform the choice of reference genes to use in the study of the sugarcane floral induction pathway. Our work also demonstrates that both *PIL5* and *LHP1* are significantly up-regulated in the initial stages of photoperiodic induction of flowering in sugarcane.

## Introduction

Sugarcane (*Saccharum* spp. hybrids) is an important economic crop worldwide, and is a predominant crop in Brazil's agribusiness mainly for sugar, ethanol, and bioelectricity production. The flowering induction process, marked by the apical meristem transition from vegetative to reproductive stage, negatively impacts sugarcane production and quality traits. Despite its economic importance, knowledge of the genes involved in the flowering induction pathway, as well as their expression patterns during the induction process, is still scarce in this high complex polyploid crop. Photoperiod is one of the major external factors that affects sugarcane flowering which can be classified as an intermediate short-day plant^1,2^. The ideal artificial photoperiodic conditions to induce flowering in sugarcane is an initial photoperiod of around 12 h and 55 min declining by 45 s per day until 12 h^3,4^.

The flowering process involves several steps governed by a complex molecular genetic network which cause changes in the apical meristem, initiating with induction up to inflorescence formation and emission. Induction, the shift to the transition from vegetative to reproductive stage promotes a change of the shoot apical meristem to an elongated dome-type structure^5^ as observed in histological sections. A greater understanding of the expression patterns of genes involved in the sugarcane flowering pathway may help identify key points of this pathway that can be manipulated to control flowering.

Reverse transcription-quantitative polymerase chain reaction (RT-qPCR) has become the most common, inexpensive and reliable method for gene expression studies. Accurate RT-qPCR analysis requires proper normalization of gene expression data through the use of reference genes whose expression should be constant in different tissues, organs and developmental stages, regardless of the experimental conditions. Until now no reference genes have been reported to be stable under all experimental conditions, the MIQE (Minimum Information for Publication of Quantitative Real-Time PCR Experiments)^6^ guidelines state that the validation of the stability of the reference gene expression patterns for different tissues under the specific conditions in which the experiment was conducted needs to be done before using them to normalize the expression levels of the target genes of interest^6–8^. The use of inappropriate reference genes can lead to errors and consequently misinterpretation of results and erroneous conclusions. In fact it is recommended to use a combination of two or more stable reference genes to get the most reliable results^9^.

There are several algorithms with mutually complementary approaches that are available for the analysis of candidate reference genes that can be used to obtain information on the best reference genes to use.

The most commonly used programs for the analysis of reference genes are BestKeeper, NormFinder, RefFinder, and GeNorm^9–12^. BestKeeper infers expression stability combining in one index those with the best geometric means, showing the correlation between each candidate reference gene and the index. The Pearson correlation (r), coefficient of determination (r^2^) and *P* value shows the relationship between candidate reference genes and the index^10^. The NormFinder algorithm provides a stability value for each gene with lower stability values indicating greater stability. This algorithm compares the relative expression of gene pairs for each sample in an experiment, and ranks the reference gene for its stability according to the repeatability of the expression difference between all samples^11^. RefFinder is an online tool (https://omictools.com/reffinder-tool) that integrates the major algorithms such as geNorm, Normfinder, BestKeeper, and the comparative Delta-Ct method to compare and sort the candidate reference genes taking into account the results of all the algorithms analyzed together generating a comprehensive ranking^12^.

To date, reference genes have not been well defined for developmental time course gene expression studies of floral induction in sugarcane, despite the relationship between flowering and yield in this crop. Few reference genes were reported for expression studies in sugarcane, such as: studies on sucrose accumulation^13^; lignin metabolism pathway^14^ and abiotic stress^15–20^, however none of these have been validated for expression studies related to flowering. Here we describe a set of reference genes with stable expression profiles evaluated in two leaf tissues (mature and immature leaf), at two time points (corresponding to the early stages of floral induction and of floral primordia formation) in a commercial sugarcane cultivar (IACSP96-7569) grown in either short day (SD) or long day (LD) photoperiods. The selected reference genes were validated by analyzing the expression of two target genes *PHYTOCHROME INTERACTING FACTOR 3-LIKE 5* (*PIL5*) and *LIKE HETEROCHROMATIN PROTEIN 1 (LHP1), involved* in the photoperiodic induction pathway^21^ and previously identified as being differentially expressed by RNA sequencing (RNA-Seq) experiment. *PIL5* is involved in the light perception pathway^22^ while *LHP1* regulates flowering time and is involved in epigenetic regulation of *FLOWERING LOCUS T* (*FT*) and *FLOWERING LOCUS C* (*FLC*) expression in *Arabidopsis thaliana*^21^.

## Results

### RT-qPCR data of candidate reference genes

After the assessment of the RT-qPCR data obtained from separate cDNA pools of samples from mature leaves and spindle leaves, primer specificity was confirmed by obtaining single melting curve peaks for all the candidate reference genes primer pairs together with the absence of any amplification in the negative controls (Supplementary Fig. S1 online). The best melting curve peaks were obtained at a primer concentration of 0.25 µM for the reference genes *UBIQUITIN 1(UBQ1)* and *TUBULIN* (*TUB)*, whilst for *UBIQUITIN 2(UBQ2)*, *GLYCERALDEHYDE-3 PHOSPHATE DEHYDROGENASE* (*GAPDH)* and *TONOPLAST INTRINSIC PROTEIN (TIPS-41)* it was at 0.4 µM, and for *60S RIBOSOMAL PROTEIN L35-4* (*RPL)*, *25S RIBOSOMAL RNA* (*25SrRNA1)* and *ELONGATION FACTOR-1Α* (*EF1)* it was at 0.8 µM (Table 1). The candidate reference gene primer pair efficiency (E) ranged from 1.76 to 2.32 in the mature leaf cDNA pool, and from 1.95 to 2.26 in the spindle leaf cDNA pool.

**Table 1:** Candidate reference genes with their respective best primer concentration, Efficiency (E) and correlation coefficient (R^2^) in pooled mature or spindle leaf cDNA samples.

Gene	Mature leaf			Spindle leaf		
	µM	E*	R^2^	µM	E*	R^2^
*TUB*	0.25	2.136	0.979	0.25	1.990	0.999
*UBQ1*	0.25	1.934	0.995	0.25	2.000	0.999
*TIPS-41*	0.4	1.765	0.999	0.4	2.034	0.999
*UBQ2*	0.4	2.147	0.997	0.4	2.075	0.997
*GAPDH*	0.4	2.323	0.999	0.4	2.264	0.995
*25SrRNA1*	0.8	2.075	0.989	0.8	2.190	0.998
*RPL*	0.8	2.174	0.999	0.8	2.048	0.999
*EF1*	0.8	2.220	0.999	0.8	1.949	0.999

### Stability analysis of candidate reference genes in different experimental samples

The stability of the candidate reference genes in different experimental samples (different tissues, developmental stage and photoperiodic conditions) was evaluated by the three algorithms, BestKeeper, NormFinder and RefFinder which complement each other and allows for a more robust analysis.

The coefficient of variation (CV) can be viewed as the first estimation of the expression stability of a reference gene. The CV determined using BestKeeper indicated that *UBQ2* is the most stable gene for spindle leaf (CV = 6.18) and SD (CV = 1.56) samples. In addition, the least variable genes were *RPL* for mature leaf (CV = 3.49), *EF1* for the 7th time point (CV = 3.00), *25SrRNA1* for the 13th time point (CV = 1.48) and *UBQ1* for LD (CV = 5.18) samples. The gene with the greater variation was *25SrRNA1* in mature leaf (CV = 22.60), spindle leaf (CV = 24.07), SD (CV = 25.55) and LD (CV = 21.13) samples. In the 7th and 13th time points the *GAPDH* gene was the least stable with CV values of 13.64 and 8.44, respectively (Table 2).

**Table 2:** Coefficient of variation (CV) according to BestKeeper algorithm, for tissue type, developmental stage (7th and 13th time points) and photoperiod treatment.

Rank	Tissue type				Developmental stage				Photoperiod treatment			
	Mature Leaf		Spindle Leaf		7th time point		13th time point		SD		LD	
	Gene	CV	Gene	CV	Gene	CV	Gene	CV	Gene	CV	Gene	CV
1	*RPL*	3.49	*UBQ2*	6.18	*EF1*	3.00	*25SrRNA1*	1.48	*UBQ2*	1.56	*UBQ1*	5.18
2	*UBQ2*	4.70	*UBQ1*	8.14	*TIPS-41*	3.40	*UBQ1*	1.78	*RPL*	3.50	*RPL*	6.44
3	*TUB*	5.41	*RPL*	8.32	*RPL*	3.64	*EF1*	4.52	*TIPS-41*	7.30	*UBQ2*	7.60
4	*TIPS-41*	6.20	*EF1*	9.72	*UBQ2*	5.22	*UBQ2*	4.76	*TUB*	7.52	*TIPS-41*	8.41
5	*UBQ1*	6.37	*TIPS-41*	9.91	*UBQ1*	5.23	*TIPS-41*	5.95	*UBQ1*	9.34	*EF1*	10.62
6	*GAPDH*	11.34	*GAPDH*	13.27	*25SrRNA1*	5.71	*TUB*	7.19	*GAPDH*	10.05	*TUB*	13.55
7	*EF1*	11.76	*TUB*	13.59	*TUB*	10.12	*RPL*	7.54	*EF1*	11.17	*GAPDH*	14.80
8	*25SrRNA1*	22.60	*25SrRNA1*	24.07	*GAPDH*	13.64	*GAPDH*	8.44	*25SrRNA1*	25.55	*25SrRNA1*	21.13

*SD* short day; *LD* long day.

The BestKeeper algorithm estimates the stability of the reference genes according to the standard deviation from the cycle threshold (Ct) raw data. Although standard deviation greater than 1 indicates inconsistent expression of the candidate reference gene^10^, De Spiegelaere et al.^23^ proposed excluding reference genes showing a standard deviation greater than 1.5. Using this calculation, the most stable genes were *UBQ2* for spindle leaf and LD samples. Although *RPL* gene was the most stable for the mature leaf, 7th time point and SD samples, it was the least stable for spindle leaf samples. *EF1* was the most stable for the 13th time point sample whereas *GAPDH* the least stable for mature leaf and SD samples (Table 3, Supplementary Table S2 online).

**Table 3:** Summarized data of the reference genes highlighted by the NormFinder and BestKeeper algorithms in different experimental samples.

Algorithms	BestKeeper				NormFinder	
Experimental samples	Gene symbol	Standard deviation	Coeff. of corr.[r]	*P* value	Gene symbol	Stability
**Mature leaf**						
Most stable gene	*RPL*	0.94	0.88	0.004	*TUB*	0.02
Least stable gene	*GAPDH*	2.91	0.18	0.669	*25SrRNA1*	0.18
Best pair	N.A	N.A	N.A	N.A	*RPL/EF1*	0.02
**Spindle leaf **						
Most stable gene	*UBQ2*	1.53	0.92	0.001	*TIPS-41*	0.04
Least stable gene	*RPL*	2.25	0.82	0.013	*25SrRNA1*	0.15
Best pair	N.A	N.A	N.A	N.A	*UBQ1/GAPDH*	0.01
**7th time point**						
Most stable gene	*RPL*	0.96	0.99	0.001	*RPL*	0.01
Least stable gene	*UBQ2*	1.27	0.34	0.418	*GAPDH*	0.09
Best pair	N.A	N.A	N.A	N.A	*UBQ1/TUB*	0.02
**13th time point**						
Most stable gene	*EF1*	1.59	0.91	0.002	*UBQ2*	0.02
Least stable gene	*UBQ1*	0.65	− 0.76	0.03	*25SrRNA1*	0.06
Best pair	*N.A*	N.A	N.A	N.A	*RPL/EF1*	0.02
**SD**						
Most stable gene	*RPL*	0.91	0.90	0.002	*UBQ1*	0.03
Least stable gene	*GAPDH*	2.48	0.35	0.393	*25SrRNA1*	0.20
Best pair	N.A	N.A	N.A	N.A	*RPL/EF1*	0.03
** LD **						
Most stable gene	*UBQ2*	1.97	0.89	0.003	*TIPS-41*	0.05
Least stable gene	*25SrRNA1*	4.25	0.98	0.001	*25SrRNA1*	0.12
Best pair	N.A	N.A	N.A	N.A	*UBQ1/TUB*	0.02

*N.A* Not applied to this algorithm. Complete data is available in Supplementary Table S2 online. Coeff.of corr. [r] stands for Pearson correlation between Bestkeeper index and candidate reference genes.

However, according to the NormFinder algorithm, *TIPS-41* was the most stable gene for two experimental samples (spindle leaf and LD) while *TUB*, *RPL*, *UBQ2* and *UBQ1* was the most stable for the mature leaf, 7th and 13th time points, and SD treatment, respectively. The least stable gene was *25SrRNA1* for six of the seven conditions while *GAPDH* was for the 7th time point (Table 3). The NormFinder algorithm also provides a combination of the two best reference genes that can be used as the most stable gene combination. *UBQ1*/*TUB* was the most stable gene combination for (7th time point and LD) and *RPL/EF1* (mature leaf, 13th time point and SD) (Table 3, Supplementary Table S2 online).

According to the RefFinder comprehensive ranking *UBQ2* was the most stable reference gene for 13th time point samples, while *TIPS-41* was for spindle leaf and LD samples, and *EF1*, *TUB* and *UBQ1* for the 7th time point, mature leaf, and SD samples, respectively (Table 4, Supplementary Tables S3 and S4 online).

**Table 4:** Summarized data from *RefFinder* Algorithm in different experimental samples.

Experimental samples			RefFinder			
Mature leaf	NormFinder		BestKeeper		Ranking	
	Gene	Stability	Gene	Stability	Gene	Stability
Most Stable Gene	*TUB*	0.623	*RPL*	0.875	*TUB*	1.316
Least Stable Gene	*25SrRNA1*	3.551	*25SrRNA1*	4.5	*25SrRNA1*	8
**Spindle Leaf**						
Most Stable Gene	*TIPS-41*	0.364	*UBQ2*	1.625	*TIPS-41*	1.57
Least Stable Gene	*UBQ1*	2.984	*25SrRNA1*	4.75	*TUB*	7.74
**7th Time point**						
Most Stable Gene	*EF1*	0.231	*EF1*	0.875	*EF1*	1.316
Least Stable Gene	*GAPDH*	3.359	*GAPDH*	3.125	*GAPDH*	8
**13th Time point**						
Most Stable Gene	*UBQ2*	0.704	*UBQ1*	0.656	*UBQ2*	1.732
Least Stable Gene	*EF1*	2.653	*TUB*	2.375	*TUB*	7.238
**SD**						
Most Stable Gene	*UBQ1*	1.614	*UBQ2*		*UBQ1*	1.565
Least Stable Gene	*25SrRNA1*	3.908	*25SrRNA1*	5	*25SrRNA1*	8
**LD**						
Most Stable Gene	*TIPS-41*	0.934	*UBQ1*	1.875	*TIPS-41*	1.414
Least Stable Gene	*EF1*	2.489	*TUB*	4.25	*TUB*	7.238

Complete data is available in Supplementary Table S3 online.

Finally, as the NormFinder algorithm also enables estimation of gene expression variation between sample subgroups of the sample set^11^ which is particularly appropriate for our experimental set-up (different tissues, time points and photoperiod treatments), greater weight was giving to the selection of the candidate reference genes by this program. Therefore, the candidate reference genes *UBQ1* and *TUB* are the best to use for normalization for most of the experimental conditions, including that in which the RNA-Seq experiment was performed (mature leaf, 7th time point and SD treatment). In addition, the pairwise *UBQ1*/*TUB* were proven as the best reference gene combination at 7th time point and LD treatment. Also *TUB* was the most stable gene at mature leaf, the tissue used for the RNA-Seq experiment.

### Validation of reference genes

The gene expression data of two target genes (*PIL5* and *LHP1*), was normalized using either the most stable (*UBQ1*/*TUB*) and least stable (*GAPDH*/*25SrRNA1*) reference gene pairs in several contrasting experimental conditions: 7th and 13th time points, SD and LD treatments, and different tissues (Mature leaf and Spindle leaf) to evaluate the effect of using good and bad reference gene pairs for normalization. Their expression was evaluated using three biological replicates, each with three technical replicates, for the two experimental conditions, assuming the inductive treatment (SD) as treated and the non-inductive treatment (LD) as the untreated, for the analysis using REST2009.

**Figure 1:**
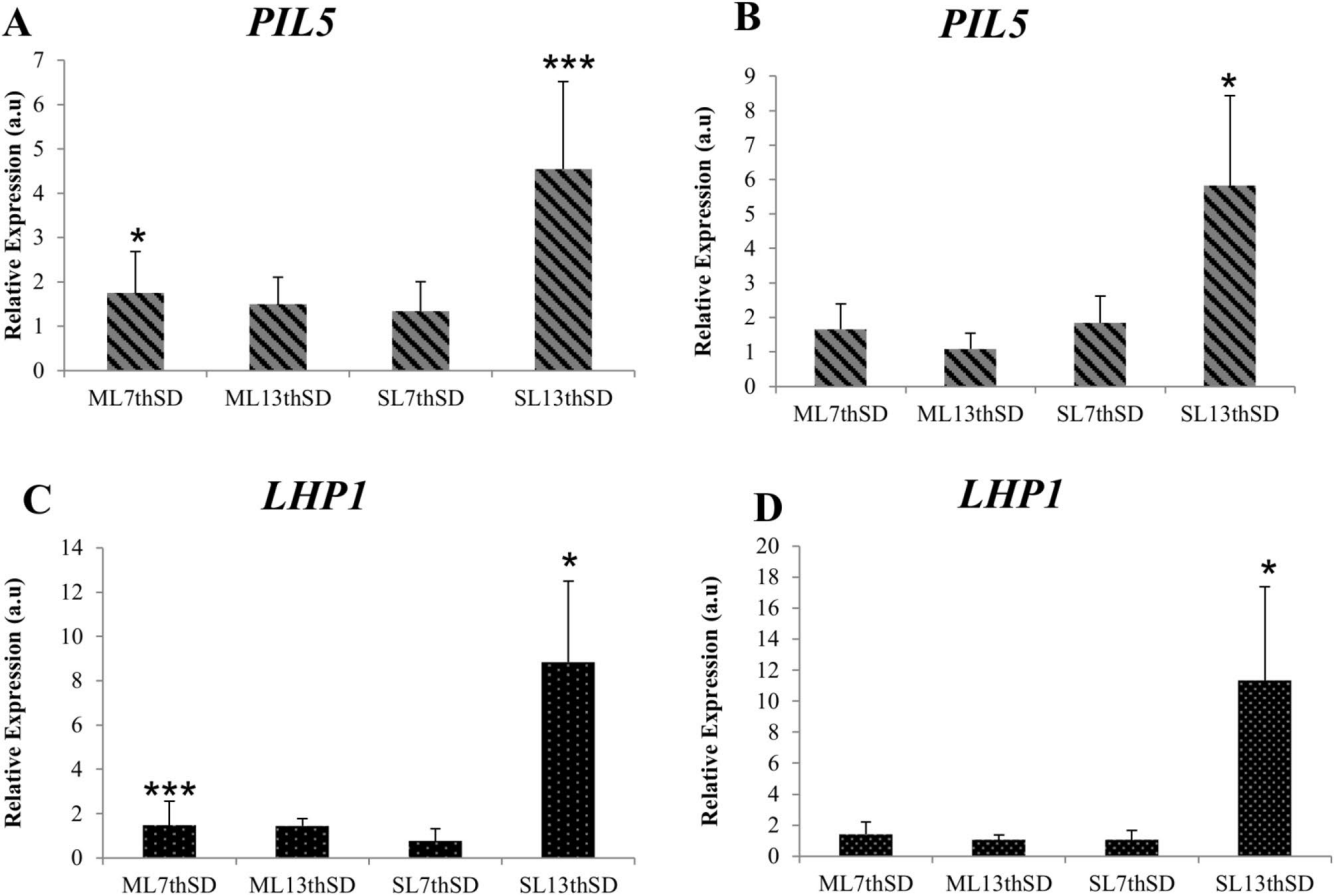
Relative expression of target genes determined by REST 2009 (n = 3). The error bars represent standard error. a.u: arbitrary units. *P* < 0.001 (***), *P* < 0.01(**), and *P* < 0.05(*). Abbreviations: *ML7th* Mature leaf 7th time point, *ML13th* Mature leaf 13th time point, *SL7th* Spindle Leaf 7th time point and *SL13th* Spindle Leaf 13th time point, *SD* short day. (**A**) *PIL5* normalized with most stable reference genes (*UBQ1*/*TUB*), (**B**) *PIL5* normalized with least stable reference genes (*GAPDH*/*25SrRNA1*), (**C**) *LHP1* normalized with most stable reference genes and (**D**) *LHP1* normalized with least stable reference genes.

In the mature leaf at the 7th time point (early stage of induction), both *PIL5* and *LHP1* were up-regulated and significantly more expressed (*P* < 0.05 and *P* < 0.001 respectively) in SD than LD when expression data was normalized with the most stable reference genes (Fig. 1A,C). However when the expression data normalization was done with the least stable reference genes (*GAPDH*/*25SrRNA1*) no significant difference was observed between SD and LD (Fig. 1B,D). In the mature leaf at the 13th time point (floral primordia stage), neither of the target genes *PIL5* and *LHP1* showed a significant difference in expression between SD and LD when normalized with either of the reference gene pairs. Similarly the spindle leaf, at the 7th time point, no significant difference in relative expression for *PIL5* and *LHP1* between SD and LD was detected. However, at the 13th time point, *PIL5* and *LHP1* were both significantly up-regulated in SD than LD samples, the relative difference in expression being large enough to be detected even when normalization is done using the least stable reference genes (Fig. 1B,D).

**Figure 2:**
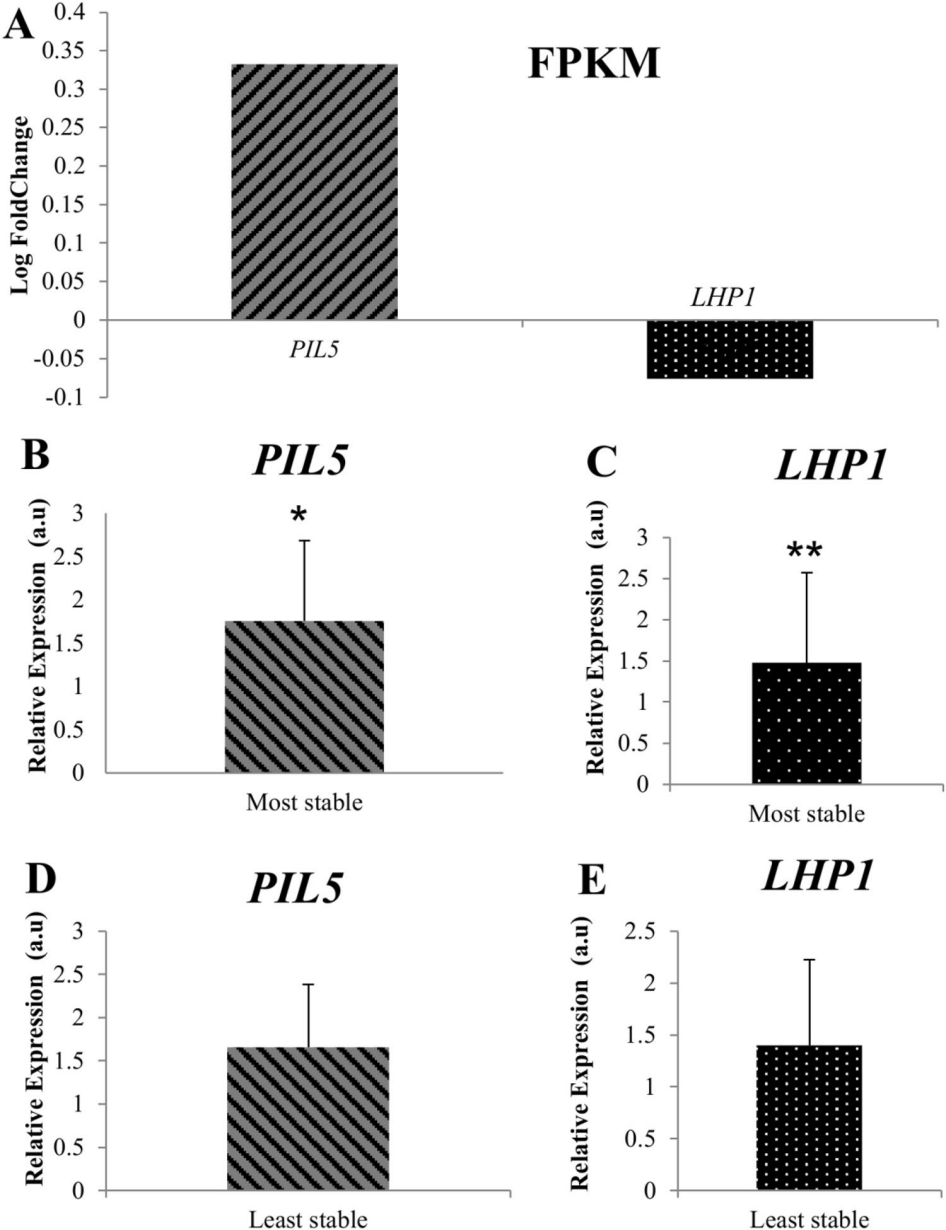
Relative expression data versus FPKM data in mature leaf 7th time point. FPKM results are represented by average from six libraries from RNA-Seq assay (*n* = 3) converted in log fold change SD/LD (**A**). Target genes normalized with the most (**B**,**C**) and least stable (**D**,**E**) reference gene pairs, the error bars, represent standard error. a.u: arbitrary units. For statistical analysis of expression levels in SD compared to LD: *P* < 0.001 (***), *P* < 0.01(**), and *P* < 0.05(*).

The expression data of the target genes given by FPKM (fragments per kilobase of exon model per million reads mapped) from the RNA-Seq was converted in log fold change (SD/LD) and compared to the RT-qPCR relative expression data, both in the 7th timepoint mature leaf samples which were used for the RNA-Seq experiment. *PIL5* had a FPKM of 2.59 and 1.20, respectively in SD and LD and a FPKM log fold change (SD/LD) of 0.33 (Fig. 2A) indicating up-regulation in SD. This gene showed a significant up- regulation with a relative expression of 1.75 by RT-qPCR only when the data was normalized with the most stable reference genes (Fig. 2B) and not with the least stable reference gene pair (Fig. 2D). FPKM values for *LHP1* at SD and LD were 33.84 and 40.36 respectively with a log fold change (SD/LD) of − 0.07 (Fig. 2A) indicating a down-regulation in SD. However, *LHP1* RT-qPCR relative expression data showed it was significantly up-regulated when normalized with the most stable reference genes (Fig. 2C), the least stable reference genes were not able to detect any significant difference (Fig. 2E).

## Discussion

Flowering is an important process beginning with the shift from vegetative to reproductive development marked by changes in the shoot apex morphology. In sugarcane, flowering takes several months to complete and can be divided into various stages each one having specific photoperiod requirements^24^. RNA-Seq and RT-qPCR are important tools to determine the expression of genes involved in the flowering process as well as to understand their role in various stages of reproduction.

The selection of reliable reference genes is an essential step in the normalization of quantitative real-time PCR gene expression data as it impacts directly on the analysis and interpretation of the RT-qPCR data.

Whilst reference genes have previously been used in the expression analysis of genes involved in sugarcane flowering, for example *GAPDH* and *ACTIN* were used to normalize the expression of *ScFT1* and *ScTFL1* in sugarcane^25^, Medeiros et al.^26^ used *EF1-α* for normalization of differentially expressed genes in early and late flowering sugarcane cultivars and Glassop et al.^2^ used *ADF* (*ACTIN DEPOLYMERISING FACTOR*) from Casu et al.^20^ as housekeeping gene to normalize genes related with photoperiod cycling over a 24-h period, these reference genes had not been experimentally validated for stability and consistency of expression between different samples at photoperiodic controlled conditions.

There are several algorithms available for the selection of reference genes, each one based on a unique set of assumptions. The use of at least three different algorithms is recommended for the validation of reference genes in qPCR assays, although, conflicting results among these algorithms can cause difficulties in the choice of the best reference genes^27^. Regarding the algorithms applied in our analyses: Bestkeeper uses standard deviation and coefficient of variation to assess the stability of the reference gene, while RefFinder integrates several different algorithms (geNorm, NormFinder and Bestkeeper) and generates a comprehensive ranking of the reference genes. However, the individual primer efficiency of each reference gene is not considered by RefFinder^23^ which assumes an efficiency of 100%. Although, BestKeeper and RefFinder can be complementarily, the ranking of stable reference genes by these algorithms in each experimental condition were not assumed to be the most appropriate option when compared to the reference genes defined by NormFinder. It is noteworthy that NormFinder algorithm not only assesses the stability of individual reference genes but also indicates the best pair of reference genes (not provided by BestKeeper and RefFinder) considering the respective primer efficiency of each gene. The most stable individual reference genes will not necessarily be selected as the best pair by the NormFinder algorithm, as selecting reference genes from the same pathway (and thus possibly co-regulated) should be avoided as the best pair^11^. In situations involving large data sets, the ANOVA of inter- and intra-groups among different conditions performed by Normfinder is more appropriate in our case for the determination of the stability values, and thus to indicate the most, and least, stable reference genes^11^.

After assessing the stability of eight different candidate reference genes using three different algorithms (BestKeeper, NormFinder and RefFinder), it was found that *UBQ1* and *TUB* were the most suitable and reliable for normalization of RT-qPCR expression data of target genes involved in the sugarcane floral induction pathway. According to the NormFinder, *TUB* and *UBQ1* stand out as the most stable genes and were observed together and in combination with other genes to compose the best reference candidate gene pairs.

Although *TIPS-41* was the most stable for immature leaf and long day conditions, it does not appear as part of a best pair in any of the conditions. This may indicate that the gene has some stability but cannot be fitted as a best pair with other candidates (due to possible co-regulation in similar molecular pathways)^27,28^. On the other hand, *TUB* and *UBQ1* were the most stable reference genes for mature leaf and short day conditions, respectively. Also, these candidates form the best pair of reference genes for the seventh time point and long day conditions. These results agree with those from RefFinder where *TUB* and *UBQ1* were the best overall candidates in the comprehensive ranking for mature leaf and short day conditions, respectively. Whether as the most stable individually, or as the best pair, *TUB/UBQ1* was able to cover the broad range of experimental conditions used in the RNA-Seq experiment (mature leaf, 7th time point, SD and LD).

The results of Bestkeeper and NormFinder, separately, together with the integrated results presented by RefFinder, point to common candidate reference genes in some experimental conditions. However, as mentioned before each algorithm uses its own intrinsic calculations. Thus, not always the most stable reference genes point out by each algorithm separately will be selected as the most stable by the RefFinder. Despite this, in the majority of the experimental conditions the different algorithms pointed to the same genes as being stable reference genes. Comparing the results from BestKeeper to that from the BestKeeper data evaluated through RefFinder (Supplementary Table S4 online), three out of six experimental conditions (mature leaf, immature leaf and LD) showed the same reference genes. In the same comparison for NormFinder, 5 out of 6 conditions (mature leaf, spindle leaf, 13th time point, SD and LD) are in agreement between the algorithms, which makes NormFinder more robust and was therefore used as our main algorithm in the selection of stable reference genes.

It is important to note that the analysis of sugarcane reference genes reported in this paper has not previously been performed over such wide experimental conditions which in this case encompasses two tissues, at two-time points in SD and LD photoperiodic treatments.

In order to demonstrate and validate the reliability of the selected reference genes they were used to normalize the expression of two target genes, *PIL5* and *LHP1*, both were differentially expressed in a previous RNA-Seq experiment performed by our research group. The expression of these two target genes was evaluated using three biological replicates, each with three technical replicates, for the three experimental conditions. By contrasting the expression data of the target genes normalized with the most stable (*UBQ1*/*TUB*) as well the least stable (*GAPDH*/*25SrRNA*) gene combination, it was verified that *UBQ1*/*TUB* are the best combination of reference genes to use for normalization as they were able to distinguish even small significant differences in gene expression (between *PIL5* and *LHP1* in mature leaves at the 7th sampling) which was not detected when using the *GAPDH*/*25SrRNA1* reference gene combination. Such small but significant differences in gene expression may be crucial in understanding physiological processes at the molecular level and underlines the need to select good reference genes and validate their expression stability in appropriate conditions before analyzing the expression of target genes.

As for the comparison between RNA-Seq expression data (FPKM) and relative expression determined by RT-qPCR, the importance of gene validation by RT-qPCR is evident not only to confirm differential gene expression (and whether it is up or down-regulated), but also the significance of the change in expression level which was only possible through the use of stable reference genes (*PIL5* up-regulation at the 7th time point on SD was confirmed as significant only when the RT-qPCR data was normalized with the most stable reference genes). The down-regulation of *LHP1* according to the RNA-Seq data was shown to be erroneous by RT-qPCR validation as it is in fact significantly up-regulated when the data is normalized using the most stable reference genes. Although both target genes (*PIL5* and *LHP1*) used to validate the selected reference genes interact with flowering regulators to control flowering time^21,29,30^, no information regarding the expression of these two target genes is available for sugarcane. Nevertheless, our RT-qPCR results suggest that *PIL5* and *LHP1* are significantly up-regulated in the initial stage of photoperiod induction, albeit functional studies in sugarcane will be necessary to determine their role in sugarcane photoperiodic induction. The reference genes *UBQ1* and *TUB* were shown to be the most stable reference genes to use to normalize RT-qPCR gene expression data during floral induction in sugarcane. On the other hand, *25SrRNA1* and *GAPDH* reference genes were the least stable in our conditions. The validation of the differential expression of genes identified by RNA-Seq analysis should be carried out by RT-qPCR relative gene expression analysis using stable reference genes for correct data interpretation.

## Methods

### Biological material and experimental conditions

IACSP96-7569 is a regular sugarcane flowering cultivar developed by the IAC Sugarcane Breeding Program with defined behavior under artificial photoperiodic regimes^4^. This cultivar was vegetatively propagated by single bud chips and planted into boxes filled with substrate (Plantmax) in a greenhouse. After 28 days, plantlets were transfer to 3.8 L tree pots (upper diameter 15.3 cm, bottom diameter 5 cm and height 35.3 cm) with equal amounts of clay soil, sand and substrate (Plantmax) and placed randomly in a photoperiod facility at the Centro de Cana–IAC at Ribeirão Preto, São Paulo, Brazil (21°12′34"S 47°52′29"W). Seven-month old plants were submitted to two different photoperiodic treatments, either a constant LD photoperiod of 13:30 h, or a SD photoperiodic treatment of 12 h and 50 min progressively shortened by 45 s per day. At weekly intervals the first visible dewlap leaf (mature leaf), immature spindle leaf, and the shoot apical meristem (SAM) were collected from three plants (biological replicates) in each photoperiodic treatment. The mature leaf and spindle leaf were immediately frozen in liquid nitrogen and later stored at − 80 °C until RNA extraction. The SAM was fixed in FAA 50% (formalin, acetic acid and ethyl alcohol) for histological sectioning to confirm the apical meristem developmental stage (see Supplementary Fig. S2 online). The time points corresponding to the 7th time point (week) (start of SAM transition to floral meristem), and 13th time point (week) sample (visible floral primordia formation), were selected for reference gene evaluation in both leaf tissues (mature leaf and leaf spindle).

### Selection of reference genes

A total of eight sugarcane candidate reference genes previously described in the literature were chosen for analysis: *UBIQUITIN1* (*UBQ1*), *UBIQUITIN2* (*UBQ2*), *60S RIBOSOMAL PROTEIN L35-4* (*RPL*), *GLYCERALDEHYDE-3-PHOSPHATE DEHYDROGENASE* (*GAPDH*), *25S RIBOSOMAL RNA* (*25SrRNA1*) and *TUBULIN* (*TUB*)^18^; *ELONGATION FACTOR 1-ALPHA* (*EF1-α*)^26^ and *TONOPLAST INTRINSIC PROTEIN* (*TIPS-41*)^15^. Two of them *GAPDH*^25^ and *EF1-α*^26^ have already been used in flowering related studies in sugarcane. Primers used for the candidate reference genes were those published and are shown in Supplementary Table S1 online. Analysis of primer efficiency was performed by RT-qPCR using a pooled cDNA sample composed of equal parts of cDNAs taken from all the spindle leaf samples (including biological replicates) and compared with a similar pool made from mature leaf samples, with the results analysed using LinRegPCR 7.5 software^31^.

Three primer concentrations (0.25, 0.4 and 0.8 µM) were used in the optimization step and the best concentration established from the melting curve peak and the primer efficiency value. Following this, the reference genes were tested in pooled cDNA samples (1:20 dilution) of three biological replicates of each sampled condition of the photoperiod experiment (see Supplementary Fig. S3 online) to allow the “in silico*”* comparison between the different experimental variables: different tissues (all mature leaf samples vs. all spindle leaf samples); developmental stage (7th vs. 13th time point); and photoperiodic conditions (SD vs. LD). The reference genes were tested by RT-qPCR and the Cycle threshold (Ct) and Efficiency (E) data were analyzed by three statistical algorithms (NormFinder, BestKeeper and RefFinder) to define the most stable reference genes. The experimental variables were analysed “in silico*”* using these algorithms (Sup.Mat.RT-qPCR data to Perform Analysis on Algorithms.xls).

### Selection of analysed genes and primer design

RNA-Seq experiment was performed on mature leaf samples from the 7th week timepoint in both SD and LD in order to find differentially expressed transcripts associated with flowering induction (data not shown).Two target genes *PIL5* and *LHP1* that are known to be differentially expressed in mature leaves at the 7th timepoint according to our RNA-Seq data were used for reference gene validation, only in 7th time point sample at mature leaf (corresponding to RNA-Seq experiment). The FPKM values from RNA- Seq data of these target genes were compared with relative expression data. The web tool PrimerQuestTool (https://www.idtdna.com/PrimerQuest/Home/Index) was used to design the primers for these two genes adopting as conditions: primer length ranging between 17 and 22 bp, GC content between 35 and 65 (%), and melting temperature (Tm) between 59º and 65 °C with an amplicon ranging between 100 and 250 bp. The primers *PIL5* and *LHP1* were designed aiming to avoid conserved domains, the web tool NetPrimer (www.premierbiosoft.com/netprimer) was used to analyze primer quality for secondary structure formation.

### Total RNA extraction and cDNA synthesis

Total RNA extraction was performed with PureLink RNA Mini Kit (ThermoFisher), quantified using a NanoDrop 2000 (Thermo Fisher Scientific, Wilmington DE, USA), and RNA integrity was verified in 1.0% agarose gel electrophoresis (3 to 5 mV/cm) stained with ethidium bromide (1 µg mL-1). All RNA samples showed 260/280 ratio of around 1.8–2.0. The cDNA synthesis was performed with QuantiNova Reverse Transcription kit (QIAGEN Strasse 1, 40724 Hilden, GERMANY) following manufacturer's instructions (genomic DNA treatment included) using 1 µg of RNA and both oligo-dT and random primers (42ºC by 15 min and 95ºC by 3 min). The cDNA was set to a final dilution of 1:20 for RT-qPCR analysis.

### RT-qPCR conditions

The RT-qPCR assays were performed using a Bio-Rad IQ5 machine using GoTaq qPCR Master Mix Kit (Promega, USA). The reaction was conducted in a final volume of 10 µl containing 5 µL of SYBR Green 2x, 3 µL of cDNA (1:20) and primer pairs at their respective adjusted concentration. Amplification conditions were: 95ºC for 3 min, followed by 40 cycles of 10 s at 95 °C and 30 s at 60ºC, followed by a melting curve from 55° to 95 °C. Both primer efficiency and concentration optimization analysis was performed using two technical replicates, whilst the evaluation of the cDNA pools under experimental conditions (tissues, time points and photoperiod treatments) as well as the validation of the reference genes were performed in triplicate (i.e. three technical replicates). Relative expression data and statistical analysis were performed using the software REST 2009 with 2000 iterations^32^ and differences considered significant when *P* < 0.001 (***), *P* < 0.01 (**), and *P* < 0.05 (*). The inductive treatment (SD) was considered “treated” and non-inductive treatment (LD) was considered “untreated” for the purpose of expression calculations in the software. For a summarized flowchart of the methods see Supplementary Fig. S4 online.

## Supplementary Information

Supplementary Information 1.

Supplementary Information 2.
