# Supplementary material for: Selection and validation of reference genes by RT-qPCR under photoperiodic induction of flowering in sugarcane (Saccharum spp.)
Source: Sci Rep. 2021 Feb 25;11:4589. doi: 10.1038/s41598-021-83918-2 (PMC7907395; doi:10.1038/s41598-021-83918-2)
Supplement: Supplementary file 2 — Supplementary Information 2. [file 41598_2021_83918_MOESM2_ESM.docx]

**Selection and Validation of Reference Genes by RT-qPCR under Photoperiodic Induction of Flowering in Sugarcane (*Saccharum* spp.).**

Paulo H. da Silva Santos, João R. Vieira Manechini, Michael S. Brito, Elisson Romanel, Renato Vicentini, Maximiliano Scarpari, Stephen Jackson and Luciana R. Pinto.

| **Gene Symbol** | **Gene name** | **Primer sequence (5´- 3´)** | **Amplicon size (bp)** | **Reference** | **Accession Number** |
| --- | --- | --- | --- | --- | --- |
| ***GAPDH*** | Glyceraldehyde-3 phosphate dehydrogenase | F: TTGGTTTCCACTGACTTCGTT  R: CTGTAGCCCCACTCGTTGT | 122 | 18 | CA254672 |
| ***TUB*** | Tubulin | F: CTCCACATTCATCGGCAACTC  R: TCCTCCTCTTCTTCCTCCTCG | 237 | 18 | CA222437 |
| ***UBQ1*** | Ubiquitin 1 | F: AGCCTCAGACCAGATTCCAA  R: AATCGCTGTCGAACTACTTGC | 110 | 18 | CA094944 |
| ***UBQ2*** | Ubiquitin 2 | F: CTTCTTCTGTCCCTCCGATG  R: TCCAACCAAACTGCTGCTC | 158 | 18 | CA093560 |
| ***RPL*** | 60S ribosomal protein L35-4 | F: CTGAAGACGGAGAGGGAAAA  R: GGCGAAGAGAAACTAACAC | 264 | 18 | CA127053 |
| ***25SrRNA1*** | 25S Ribosomal RNA | F: ATAACCGCATCAGGTCTCCAAG  R: CCTCAGAGCCAATCCTTTTCC | 110 | 18 | CA171131/ CO373883 |
| ***EF1α*** | Elongation factor-1α | F: AAGGCCCGTTATGATGAGTTGTG  R: CAAAACCAGAGATTGGGACGAAAG | 100 | 23 | AF331850 |
| ***TIPS-41*** | Tonoplast intrinsic protein | F: CACCTGTTGAGGTTCCTGCT  R: CACAGCATCACTCCCACAGT | 116 | 15 | CA228782.1 |
| ***PIF1*** | Phytochrome interacting factor 3-like 5 | F: GGTCTGGCTCTGCATGAC  R: AACCAGTTCGTCCCTGATTG | 117 | This paper |  |
| ***LHP1*** | Like heterochromatin protein | F: CTCCTTGCTCCTGTGTTACTTC  R: GCACCCTGCTAATGGTTCAT | 143 | This paper |  |

**Supplementary Table S1.** Reference and target genes with respective primer sequences and amplicon size in base pairs (bp).

**Supplementary Table S2.** Data from the NormFinder and BestKeeper algorithms under different experimental conditions.

| **Experimental** | ***NormFinder*** | | ***BestKeeper*** | | |
| --- | --- | --- | --- | --- | --- |
| **Sample** |  |  |  |  |  |
| Mature Leaf | Gene | Stability | Gene | Standard deviation | coeff. of corr. [r] |
|  | *TUB* | 0.02 | *RPL* | 0.94 | 0.55 |
|  | *UBQ1* | 0.04 | *UBQ2* | 1.23 | 0.81 |
|  | *TIPS-41* | 0.04 | *TUB* | 1.66 | 0.87** |
|  | *EF1* | 0.06 | *TIPS-41* | 1.9 | 0.97*** |
|  | *UBQ2* | 0.06 | *UBQ1* | 2.18 |  |
|  | *RPL* | 0.08 | *GAPDH* | 2.91 | 0.18 |
|  | *GAPDH* | 0.11 | *EF1* | 3.86 | 0.98*** |
|  | *25SrRNA1* | 0.18 | *25SrRNA1* | 4.59 | 0.35 |
| Best Pair | *RPL/EF1* | 0.02 | *N.A* | N.A | N.A |
| Most stable Gene | *TUB* | 0.02 | *RPL* | 0.94 | 0.55 |
| Spindle Leaf | *TIPS-41* | 0.04 | *UBQ2* | 1.53 | 0.81** |
|  | *EF1* | 0.04 | *RPL* | 2.25 | 0.82** |
|  | *GAPDH* | 0.06 | *UBQ1* | 2.78 | 0.84** |
|  | *UBQ1* | 0.06 | *EF1* | 2.98 | 0.89** |
|  | *RPL* | 0.06 | *TIPS-41* | 3.16 | 0.96*** |
|  | *UBQ2* | 0.07 | *GAPDH* | 3.14 | 0.95*** |
|  | *TUB* | 0.08 | *TUB* | 3.73 | 0.92*** |
|  | *25SrRNA1* | 0.15 | *25SrRNA1* | 4.74 | 0.96*** |
| Best Pair | *UBQ1/GAPDH* | 0.01 | *N.A* | N.A | N.A |
| Most stable Gene | *TIPS-41* | 0.04 | *UBQ2* | 1.53 | 0.81** |
| 7^th^ timepoint sample | *RPL* | 0.01 | *UBQ2* | 1.27 | 0.34 |
|  | *EF1* | 0.03 | *TIPS-41* | 0.99 | 0.56 |
|  | *TIPS-41* | 0.05 | *EF1* | 0.85 | 0.85** |
|  | *UBQ1* | 0.05 | *UBQ1* | 1.66 | 0.55 |
|  | *25SrRNA1* | 0.05 | *25SrRNA1* | 0.88 | 0.65 |
|  | *TUB* | 0.07 | *RPL* | 0.96 | 0.99*** |
|  | *UBQ2* | 0.08 | *TUB* | 2.67 | 0.84** |
|  | *GAPDH* | 0.09 | *GAPDH* | 3.16 | 0.85** |
| Best Pair | *UBQ1/TUB* | 0.02 | *N.A* | N.A | N.A |
| Most stable Gene | *RPL* | 0.01 | *UBQ2* | 1.27 | 0.34 |
| 13^th^ timepoint sample | *UBQ2* | 0.02 | *UBQ1* | 0.65 | -0.76 |
|  | *RPL* | 0.05 | *25SrRNA1* | 0.37 | -0.66 |
|  | *TIPS-41* | 0.06 | *EF1* | 1.59 | 0.067 |
|  | *TUB* | 0.06 | *UBQ2* | 1.26 | 0.94*** |
|  | *EF1* | 0.06 | *TIPS-41* | 1.99 | 0.64 |
|  | *GAPDH* | 0.06 | *TUB* | 2.28 | 0.83** |
|  | *UBQ1* | 0.06 | *GAPDH* | 2.21 | 0.96*** |
|  | *25SrRNA1* | 0.06 | *RPL* | 2.09 | 0.89** |
| Best Pair | *RPL/EF1* | 0.02 | *N.A* | N.A | N.A |
| Most stable Gene | *UBQ2* | 0.02 | *UBQ1* | 0.65 | -0.76 |
| SD photoperiod | *UBQ1* | 0.03 | *RPL* | 0.91 | 0.27 |
|  | *EF1* | 0.05 | *UBQ2* | 0.39 | 0.67 |
|  | *TUB* | 0.05 | *GAPDH* | 2.48 | 0.35 |
|  | *TIPS-41* | 0.06 | *TUB* | 2.12 | 0.79* |
|  | *UBQ2* | 0.07 | *TIPS-41* | 2.21 | 0.75* |
|  | *RPL* | 0.08 | *EF1* | 3.5 | 0.94*** |
|  | *GAPDH* | 0.11 | *UBQ1* | 3.19 | 0.98*** |
|  | *25SrRNA1* | 0.2 | *25SrRNA1* | 5.08 | 0.91** |
| Best Pair | *RPL/EF1* | 0.03 | *N.A* | N.A | N.A |
| Most stable Gene | *UBQ1* | 0.03 | *RPL* | 0.91 | 0.27 |
| LD photoperiod | *TIPS-41* | 0.05 | *UBQ1* | 1.77 | 0.81** |
|  | *GAPDH* | 0.05 | *UBQ2* | 1.97 | 0.81** |
|  | *EF1* | 0.06 | *RPL* | 1.81 | 0.83** |
|  | *RPL* | 0.06 | *TIPS-41* | 2.71 | 0.94*** |
|  | *UBQ1* | 0.06 | *EF1* | 3.4 | 0.85** |
|  | *UBQ2* | 0.06 | *GAPDH* | 3.66 | 0.99*** |
|  | *TUB* | 0.07 | *TUB* | 4.06 | 0.94*** |
|  | *25SrRNA1* | 0.12 | *25SrRNA1* | 4.25 | 0.98*** |
| Best Pair | *UBQ1/TUB* | 0.02 | *N.A* | N.A | N.A |
| Most stable Gene | *TIPS-41* | 0.05 | *UBQ1* | 1.77 | 0.81** |

N.A: Not applied to this algorithm. P <0.001 (***), P<0.01(**), and P<0.05(*).

**Supplementary Table** **S3.** *RefFinder* Algorithm Data.

| **Experimental Condition** | **RefFinder** | | | | | | | | | |
| --- | --- | --- | --- | --- | --- | --- | --- | --- | --- | --- |
| Mature Leaf | **NormFinder** | | **BestKeeper** | | **GeNorm** | | **Delta CT** |  | **Ranking** | |
|  | Gene | Stability | Gene | Stability | Gene | Estab. | Gene | Stability | Gene | Stability. |
|  | *TUB* | 0.62 | *RPL* | 0.875 | *TUB* | 1.246 | *TUB* | 2.157 | *TUB* | 1.316 |
|  | *TIPS-41* | 0.99 | *UBQ2* | 1.281 | *TIPS-41* | 1.246 | *TIPS-41* | 2.446 | *TIPS-41* | 2 |
|  | *UBQ1* | 1.49 | *TUB* | 1.75 | *UBQ2* | 1.647 | *UBQ1* | 2.698 | *UBQ2* | 3.13 |
|  | *UBQ2* | 1.91 | *TIPS-41* | 1.938 | *RPL* | 1.728 | *UBQ2* | 2.8 | *RPL* | 3.162 |
|  | *RPL* | 2.13 | *UBQ1* | 2.125 | *UBQ1* | 2.013 | *RPL* | 2.864 | *UBQ1* | 3.873 |
|  | *EF1* | 2.37 | *GAPDH* | 2.875 | *EF1* | 2.352 | *EF1* | 3.089 | *EF1* | 6.236 |
|  | *GAPDH* | 3.42 | *EF1* | 3.75 | *GAPDH* | 2.671 | *GAPDH* | 3.816 | *GAPDH* | 6.735 |
|  | *25SrRNA1* | 3.55 | *25SrRNA1* | 4.5 | *25SrRNA1* | 2.964 | *25SrRNA1* | 3.846 | *25SrRNA1* | 8 |
| Best Pair | N.A | N.A | N.A | N.A | N.A | N.A | N.A | N.A | N.A | N.A |
| Best gene | *TUB* | 0.623 | *RPL* | 0.875 | *TIPS-41* | 1.246 | *TUB* | 2.157 | *TUB* | 1.316 |
| Spindle Leaf | *TIPS-41* | 0.364 | *UBQ2* | 1.625 | *EF1* | 1.188 | *TIPS-41* | 2.03 | *TIPS-41* | 1.57 |
|  | *GAPDH* | 1.512 | *RPL* | 2.375 | *TIPS-41* | 1.188 | *GAPDH* | 2.43 | *EF1* | 2.45 |
|  | *EF1* | 1.727 | *UBQ1* | 3 | *UBQ1* | 1.21 | *EF1* | 2.45 | *GAPDH* | 3.16 |
|  | *UBQ2* | 1.912 | *EF1* | 3 | *25SrRNA1* | 1.59 | *UBQ1* | 2.64 | *UBQ2* | 3.31 |
|  | *RPL* | 2.187 | *GAPDH* | 3.125 | *GAPDH* | 2.11 | *UBQ2* | 2.66 | *UBQ1* | 3.66 |
|  | *25SrRNA1* | 2.375 | *TIPS-41* | 3.125 | *UBQ2* | 2.314 | *RPL* | 2.82 | *RPL* | 4.74 |
|  | *TUB* | 2.606 | *TUB* | 3.75 | *RPL* | 2.476 | *25SrRNA1* | 2.91 | *25SrRNA1* | 6.29 |
|  | *UBQ1* | 2.984 | *25SrRNA1* | 4.75 | *TUB* | 2.626 | *TUB* | 3.08 | *TUB* | 7.74 |
| Best Pair | N.A | N.A | N.A | N.A | N.A | N.A | *N.A* | N.A | *N.A* | N.A |
| Best gene | *TIPS-41* | 0.364 | *UBQ2* | 1.625 | *EF1* | 1.188 | *TIPS-41* | 2.03 | *TIPS-41* | 1.57 |
| 7th Timepoint | *RPL* | 0.231 | *EF1* | 0.875 | *25SrRNA1* | 0.518 | *EF1* | 1.557 | *EF1* | 1.316 |
|  | *EF1* | 0.231 | *25SrRNA1* | 0.938 | *TIPS-41* | 0.518 | *RPL* | 1.582 | *25SrRNA1* | 2.213 |
|  | *TIPS-41* | 1.149 | *RPL* | 1 | *EF1* | 0.622 | *25SrRNA1* | 1.782 | *RPL* | 2.632 |
|  | *25SrRNA1* | 1.207 | *TIPS-41* | 1 | *RPL* | 0.743 | *TIPS-41* | 1.792 | *TIPS-41* | 2.632 |
|  | *UBQ1* | 1.816 | *UBQ2* | 1.156 | *UBQ1* | 0.949 | *UBQ1* | 2.223 | *UBQ1* | 5.233 |
|  | *UBQ2* | 1.847 | *UBQ1* | 1.75 | *UBQ2* | 1.367 | *UBQ2* | 2.427 | *UBQ2* | 5.733 |
|  | *TUB* | 2.019 | *TUB* | 2.625 | *TUB* | 1.73 | *TUB* | 2.598 | *TUB* | 7 |
|  | *GAPDH* | 3.359 | *GAPDH* | 3.125 | *GAPDH* | 2.193 | *GAPDH* | 3.581 | *GAPDH* | 8 |
| Best Pair | N.A | N.A | N.A | N.A | N.A | N.A | N.A | N.A | N.A | N.A |
| Best Gene | *EF1* | 0.231 | *EF1* | 0.875 | *TIPS-41* | 0.518 | *EF1* | 1.557 | *EF1* | 1.316 |
| 13thTimepoint | *UBQ2* | 0.704 | *25SrRNA1* | 0.219 | *RPL* | 1.302 | *UBQ2* | 1.872 | *UBQ2* | 1.732 |
|  | *GAPDH* | 1.346 | *UBQ1* | 0.656 | *GAPDH* | 1.302 | *GAPDH* | 2.224 | *GAPDH* | 2.3 |
|  | *25SrRNA1* | 1.413 | *UBQ2* | 1.125 | *UBQ2* | 1.546 | *25SrRNA1* | 2.264 | *25SrRNA1* | 2.59 |
|  | *RPL* | 1.825 | *EF1* | 1.625 | *TIPS-41* | 1.786 | *RPL* | 2.467 | *RPL* | 3.13 |
|  | *TIPS-41* | 2.05 | *TIPS-41* | 2 | *25SrRNA1* | 2.038 | *UBQ1* | 2.625 | *UBQ1* | 4.356 |
|  | *UBQ1* | 2.058 | *RPL* | 2.156 | *UBQ1* | 2.162 | *TIPS-41* | 2.675 | *TIPS-41* | 4.949 |
|  | *TUB* | 2.362 | *GAPDH* | 2.25 | *TUB* | 2.315 | *TUB* | 2.85 | *EF1* | 6.727 |
|  | *EF1* | 2.653 | *TUB* | 2.375 | *EF1* | 2.508 | *EF1* | 3.085 | *TUB* | 7.238 |
| Best Pair | N.A | N.A | N.A | N.A | N.A | N.A | N.A | N.A | N.A | N.A |
| Best gene | *UBQ2* | 0.704 | *UBQ1* | 0.656 | *RPL* | 1.302 | *UBQ2* | 1.872 | *UBQ2* | 1.732 |
| SD photoperiod | *UBQ1* | 1.614 | *UBQ2* | 0.625 | *UBQ1* | 1.035 | *UBQ1* | 2.868 | *UBQ1* | 1.565 |
|  | *UBQ2* | 1.693 | *RPL* | 0.875 | *EF1* | 1.035 | *UBQ2* | 2.934 | *UBQ2* | 2 |
|  | *TUB* | 1.769 | *TIPS-41* | 2.094 | *TIPS-41* | 2.181 | *EF1* | 2.995 | *EF1* | 3.201 |
|  | *TIPS-41* | 1.93 | *TUB* | 2.25 | *UBQ2* | 2.551 | *TUB* | 3.062 | *TIPS-41* | 3.834 |
|  | *EF1* | 1.984 | *GAPDH* | 2.375 | *TUB* | 2.695 | *RPL* | 3.144 | *TUB* | 3.936 |
|  | *RPL* | 2.306 | *UBQ1* | 3.25 | *RPL* | 2.75 | *TIPS-41* | 3.15 | *RPL* | 4.356 |
|  | *GAPDH* | 3.604 | *EF1* | 3.5 | *GAPDH* | 3.001 | *GAPDH* | 4.047 | *GAPDH* | 6.435 |
|  | *25SrRNA1* | 3.908 | *25SrRNA1* | 5 | *25SrRNA1* | 3.299 | *25SrRNA1* | 4.196 | *25SrRNA1* | 8 |
| Best Pair | N.A | N.A | N.A | N.A | N.A | N.A | N.A | N.A | N.A | N.A |
| Best gene | *UBQ1* | 1.614 | *UBQ2* |  | *UBQ1* | 1.035 | *UBQ1* | 2.868 | *UBQ1* | 1.565 |
| LD photoperiod | *TIPS-41* | 0.934 | *UBQ1* | 1.875 | *UBQ1* | 1.195 | *TIPS-41* | 2.121 | *TIPS-41* | 1.414 |
|  | *GAPDH* | 1.143 | *UBQ2* | 1.969 | *TIPS-41* | 1.195 | *GAPDH* | 2.223 | *UBQ1* | 2.236 |
|  | *25SrRNA1* | 1.646 | *RPL* | 1.969 | *RPL* | 1.593 | *25SrRNA1* | 2.46 | *GAPDH* | 3.31 |
|  | *RPL* | 1.903 | *TIPS-41* | 2.875 | *25SrRNA1* | 1.999 | *RPL* | 2.59 | *RPL* | 3.464 |
|  | *UBQ1* | 1.968 | *EF1* | 3.375 | *GAPDH* | 2.108 | *UBQ1* | 2.595 | *25SrRNA1* | 3.984 |
|  | *UBQ2* | 2.062 | *GAPDH* | 3.75 | *UBQ2* | 2.28 | *UBQ2* | 2.753 | *UBQ2* | 4.559 |
|  | *TUB* | 2.456 | *25SrRNA1* | 4.25 | *TUB* | 2.442 | *TUB* | 2.944 | *EF1* | 7.113 |
|  | *EF1* | 2.489 | *TUB* | 4.25 | *EF1* | 2.59 | *EF1* | 3.033 | *TUB* | 7.238 |
| Best Pair | N.A | N.A | N.A | N.A | N.A | N.A | N.A | N.A | N.A | N.A |
| Best gene | *TIPS-41* | 0.934 | *UBQ1* | 1.875 | *UBQ1* | 1.195 | *TIPS-41* | 2.121 | *TIPS-41* | 1.414 |

N.A: Not applied to this algorithm.

**Supplementary Table** **S4.** Comparison between the stability data of the individual algorithms and the RefFinder with agreements between NormFinder and BestKeeper when compared individually and by RefFinder.

|  | **Individually** | | | | **RefFinder** | | | |
| --- | --- | --- | --- | --- | --- | --- | --- | --- |
| **Experimental condition** | **NormFinder** | | **BestKeeper** | | **NormFinder** | | **BestKeeper** | |
| **Mature Leaf** | Gene | Stability | Gene | Stability | Gene | Stability | Gene | Stability |
| Most stable gene | *TUB* | 0.02 | *RPL* | 1.30 | *TUB* | 0.623 | *RPL* | 0.875 |
| Least stable gene | *25SrRNA1* | 0.18 | *25SrRNA1* | 1.60 | *25SrRNA1* | 3.55 | *25SrRNA1* | 4.5 |
| **Spindle Leaf** |  |  |  |  |  |  |  |  |
| Most stable gene | *TIPS-41* | 0.04 | *UBQ2* | 1.41 | *TIPS-41* | 0.364 | *UBQ2* | 1.625 |
| Least stable gene | *25SrRNA1* | 0.15 | *25SrRNA1* | 3.41 | *UBQ1* | 2.984 | *25SrRNA1* | 4.75 |
| **7th Time point** |  |  |  |  |  |  |  |  |
| Most stable gene | *RPL* | 0.01 | *RPL* | 0.91 | *EF1* | 0.231 | *EF1* | 0.875 |
| Least stable gene | *GAPDH* | 0.09 | *GAPDH* | 5.50 | *GAPDH* | 3.359 | *GAPDH* | 3.125 |
| **13th Time point** |  |  |  |  |  |  |  |  |
| Most stable gene | *UBQ2* | 0.02 | *EF1* | 1.59 | *UBQ2* | 0.704 | *UBQ1* | 0.656 |
| Least stable gene | *25SrRNA1* | 0.06 | *RPL* | 4.65 | *EF1* | 2.653 | *TUB* | 2.375 |
| **Short day** |  |  |  |  |  |  |  |  |
| Most stable gene | *UBQ1* | 0.03 | *RPL* | 1.10 | *UBQ1* | 1.614 | *UBQ2* |  |
| Least stable gene | *25SrRNA1* | 0.20 | *25SrRNA1* | 6.98 | *25SrRNA1* | 3.908 | *25SrRNA1* | 5 |
| **Long day** |  |  |  |  |  |  |  |  |
| Most stable gene | *TIPS-41* | 0.05 | *UBQ1* | 1.41 | *TIPS-41* | 0.934 | *UBQ1* | 1.875 |
| Least stable gene | *25SrRNA1* | 0.12 | *25SrRNA1* | 3.06 | *EF1* | 2.489 | *TUB* | 4.25 |

**
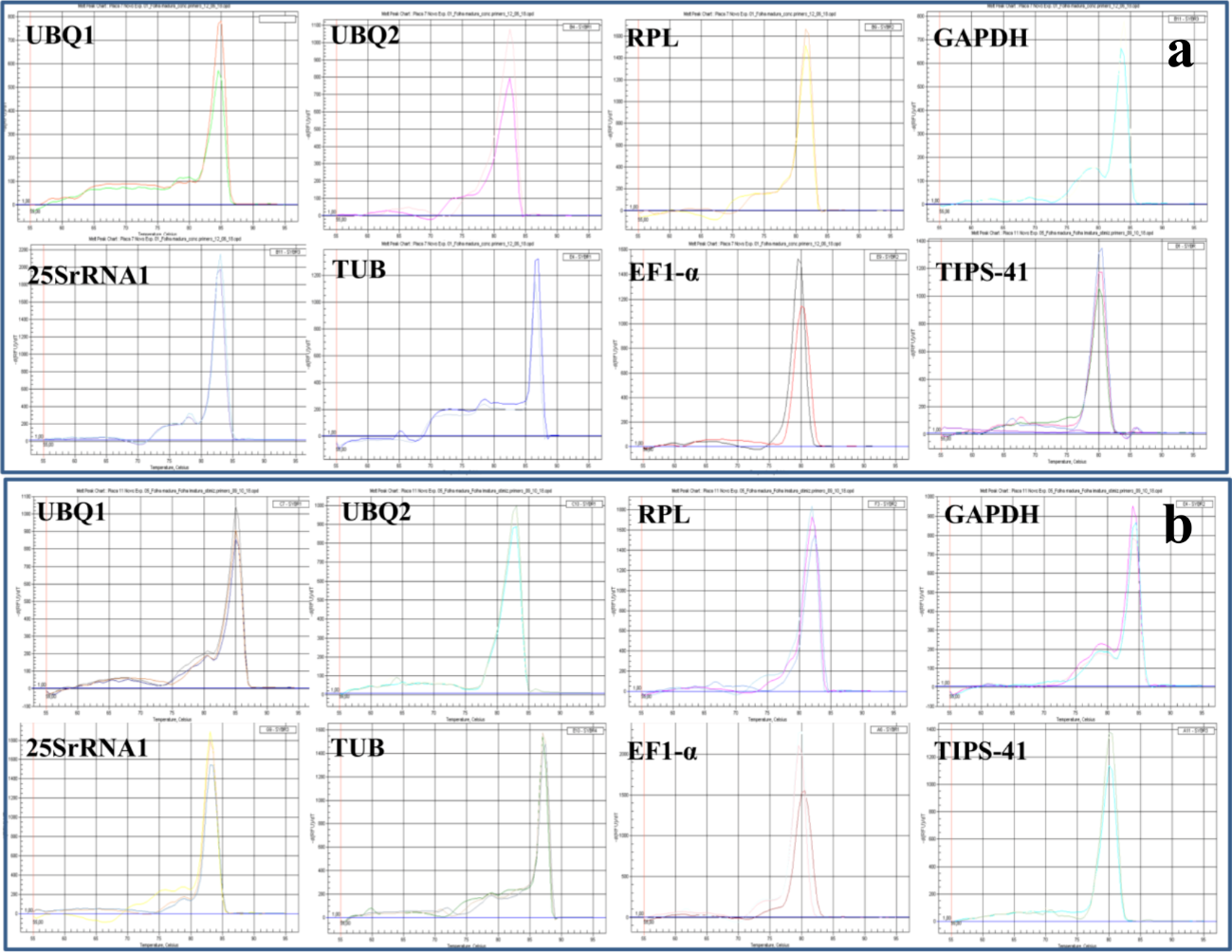
**

**Supplementary Figure S1.** Mature leaf and spindle leaf melt curve peaks from candidates to reference genes. (a) Melt curve peaks from a technical duplicate RT-qPCR of a mature leaf sample and (b) Melt curve peaks from a technical duplicate RT-qPCR of a spindle leaf sample.

**
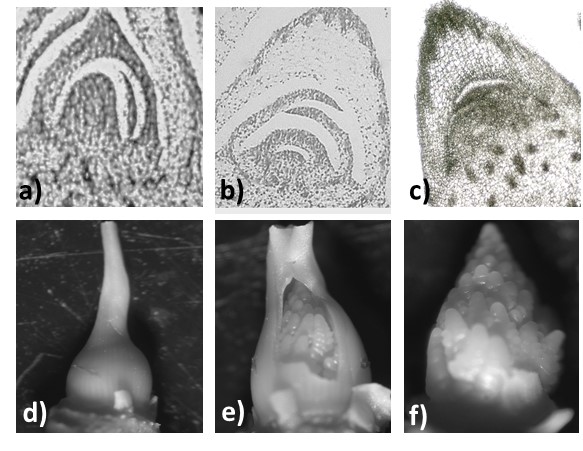
**

**Supplementary Figure S2.** Morphology of the shoot apical meristem of sugarcane cultivar IACSP96-7569 at different stages of photoperiodic induction. a) 7^th^ week, 45 days in inductive condition (SD); b) 7^th^ week, 45 days in non-inductive condition (LD); c) 13^th^  week, 85 days in non-inductive condition (LD); d); e); f) 13^th^ week, 85 days in inductive condition (SD).


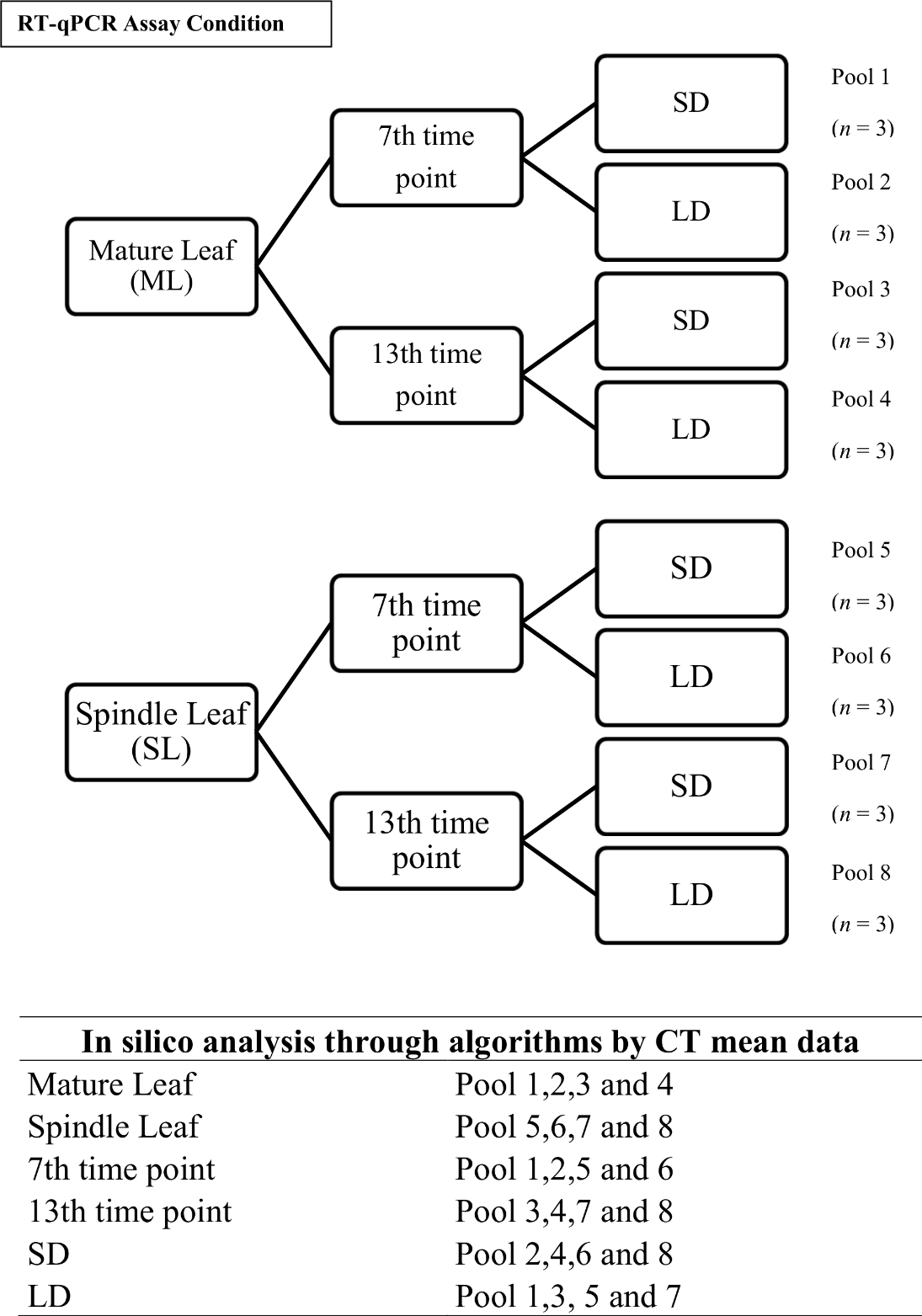


**Supplementary Figure S3.** Diagram of the *in silico* analysis by algorithms based on the cycle threshold experimental samples. Triplicate samples were harvested for each sample giving rise to each pool. Abbreviations: ML: Mature leaf, SL: Spindle Leaf, 7^th^: 7^th^ timepoint, 13^th^: 13^th^ timepoint, SD: Short day and LD: Long day.


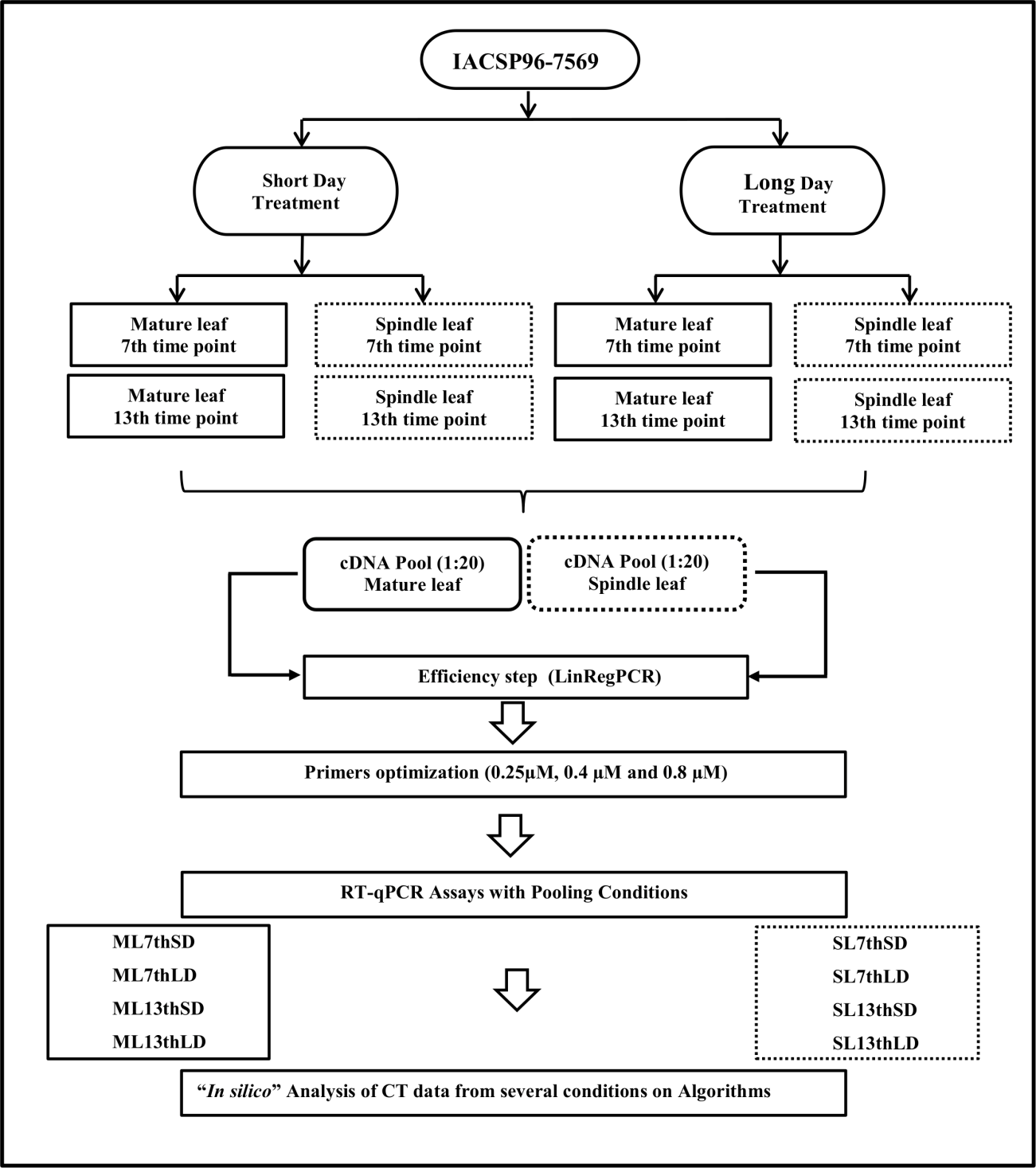


**Supplementary Figure S4.** Flowchart summarizing experimental setup. The IACSP96-7569 cultivar was submitted to two different photoperiodic treatments. Mature leaf and spindle leaf was sampled at two time points. Two separate cDNA pools (mature leaf and spindle leaf) were used for primer efficiency and optimization. RT-qPCR was performed on cDNA pools of three biological replicates from each sampled conditions. Based on the Ct value of the cDNA pools, an “*in silico”* analysis was performed for each specific condition by the three algorithms.
